# Postprandial glycemic response in a non-diabetic adult population: the effect of nutrients is different between men and women

**DOI:** 10.1186/s12986-019-0368-1

**Published:** 2019-07-17

**Authors:** María González-Rodríguez, Marcos Pazos-Couselo, José M. García-López, Santiago Rodríguez-Segade, Javier Rodríguez-García, Carmen Túñez-Bastida, Francisco Gude

**Affiliations:** 1 Department of Endocrinology and Nutrition, Complejo Hospitalario Universitario de Santiago de Compostela, Travesía da Choupana, s/n, 15706 Santiago de Compostela, Spain; 2 Psychiatry, Radiology and Public Health Department, University of Santiago de Compostela, Santiago de Compostela, Spain; 3 Department of Biochemistry and Molecular Biology, University of Santiago de Compostela, Santiago de Compostela, Spain; 4 Primary Care Center of A Estrada (CTB), Santiago de Compostela, Spain; 5 Clinical Epidemiology Unit, Complejo Hospitalario Universitario de Santiago de Compostela, Santiago de Compostela, Spain

**Keywords:** Postprandial glycemic response, Continuous glucose monitoring, Non-diabetic population, Healthy population

## Abstract

**Background:**

There is a growing interest in the pathopysiological consequences of postprandial hyperglycemia. It is well known that in diabetic patients 2 h plasma glucose is a better risk predictor for coronary heart disease than fasting plasma glucose. Data on the glycemic response in healthy people are scarce.

**Objective:**

To evaluate the effect of macronutrients (carbohydrates, fats, and proteins) and fiber on postprandial glycemic response in an observational study of a non-diabetic adult population.

**Design:**

Cross-sectional study. 150 non-diabetic adults performed continuous glucose monitoring for 6 days. During this period they recorded food and beverage intake. The participants were instructed not to make changes in their usual diet and physical exercise.

Variables analyzed included clinical parameters (age, sex, body weight, height, body mass index, blood pressure, and waist measurement), meal composition (calories, carbohydrates, fats, proteins, and fiber) and glycemic postprandial responses separated by sexes.

The study period was defined from the start of dinner to 6 h later.

**Results:**

A total of 148 (51% women) subjects completed all study procedures. Dinner intake was higher in males than in females (824 vs 531 kcal). Macronutrient distribution was similar in both sexes. No significant differences were found in fiber intake between men and women (5.5 g vs 4.5 g).

In both sexes, the higher intake of carbohydrates corresponded to a significantly higher glycemic response (*p* = 0.0001 in women, *p* = 0.022 in men). Moreover, in women, as fat intake was higher, a flattening of the postprandial glycemic curve was observed (*p* = 0.003). With respect to fiber, a significantly lower glycemic response was observed in the group of women whose fiber intake at dinner was higher (*p* = 0.034).

**Conclusions:**

Continuous glucose monitoring provides important information about glucose levels after meals. In this study, the postprandial glycemic response in women was different from that of men, and carbohydrates were the main determinant of elevated postprandial glucose levels.

## Background

There is a growing interest in the pathopysiological consequences of postprandial hyperglycemia. It is well known that in diabetic patients 2 h plasma glucose is a better risk predictor for coronary heart disease than fasting plasma glucose [1]. Also normoglycemic subjects with higher levels of 2 h plasma glucose had higher risk of devoloping diabetes [2].

Clinical trials have shown the importance of maintaining blood glucose levels after meals within the normal range in patients with type 2 diabetes to prevent its complications and mortality [1, 3, 4].

The glycemic response to meals has been studied widely in patients with diabetes mellitus, especially in type 1 [5–12]. However, data on the glycemic response to meals in healthy people are scarce. In most previous works, the population samples were small and capillary blood samples were used to analyze glucose levels, resulting in a limited view of the glycemic patterns in free-living conditions. The previous studies were focused on the influence of some nutrient or food on glycemic response, and they were conducted in controlled conditions or during hospitalization. Few studies have considered the pattern of regular intake that is typically part of daily life, and they do not use continuous glucose monitoring (CGM) as a blood glucose measurements system [13–16].

Continuous monitoring of interstitial blood glucose provides an opportunity to better understand the alterations of glucose metabolism in healthy individuals. It is currently recognized as a good tool to identify glycemic excursions in patients with type 1 and type 2 diabetes mellitus. However, few data are available regarding CGM in healthy people [17]. Unlike capillary blood glucose meters, CGM provides information about a period of time and can even give information in “real time” about the glucose value, speed, and direction. The inability to detect glycemic fluctuations is another limitation of traditional capillary glycemia.

Glycemic fluctuations in the non-diabetic population are closely controlled by physiological mechanisms. Dietary factors also influence glycemic excursions, especially during the postprandial period. Therefore, dietary interventions represent an important strategy to attenuate these oscillations and improve postprandial glycemia.

The aim of this study was to evaluate the effect of different macronutrients (carbohydrates, fats, and proteins) and fiber on postprandial glycemic response in an observational study of a non-diabetic adult population.

## Subjects and methods

The present work was carried out within the epidemiological study “A Estrada Glycation and Inflammation Study” (AEGIS; trial NCT01796184 at https://clinicaltrials.gov/ct2/show/NCT01796184?term=NCT01796184&rank=1), a cross-sectional study that was performed in the municipality of A Estrada, in Northwestern Spain. An age-stratified random sample of the population aged 18 years and older was drawn from Spain’s National Health System Registry. A total of 1516 subjects agreed to participate in the study, which comprised an interviewer-administered structured questionnaire that included demographic and anthropometric data, a lifestyle description (physical exercise, alcohol consumption and smoking), and fasting venous blood sampling. In addition, 622 study subjects consented to undergo a 6-day period of CGM [18].

A subsample was taken from the total of monitored patients. The first 150 individuals (75 men and 75 women) who met the following criteria were included: (a) not previously diagnosed with type 2 diabetes, (b) glycated hemoglobin < 6.5% and/or a fasting blood glucose < 126 mg/dL, (c) ability to perform CGM system, and (d) ability to provide informed written consent.

The protocol was as follows: All subjects were successively convened for one day in the Primary Care Center for evaluation, including: (a) an interviewer-administered structured questionnaire that included demographic and anthropometric data, (b) fasting venous blood sampling, and (c) a 6–day period of CGM.

### CGM procedures

At the start of each monitoring period, a research nurse inserted a sensor (Enlite™ Medtronic) subcutaneously into the abdomen of each participant and instructed them in the use of the device (iPro™ 2 Medtronic). The sensor continuously measures interstitial glucose levels in the subcutaneous tissue, recording values every 5 min, within a range of 40 to 400 mg/dL (2.2–22.2 mmol/L). Participants were provided with a conventional glucometer (One Touch Verio Pro; LifeScan, Milpitas, CA, USA) as well as compatible lancets and test strips for calibrating the CGM device. Subjects were asked to make at least 3 capillary blood glucose measurements (before the main meals).

During the CGM period, participants also monitored their intake of food and beverages.

### Dietary record

The individuals recorded in detail everything they ate and drank, indicating the amounts of food and drinks ingested during the monitoring period, preparation mode (ingredients, cooking method, sauces, brand of the products) and meal times.

The participants were instructed not to make changes in their usual diet or their daily routine of work and physical exercise.

At the end of the monitored period, coinciding with the removal of the glucose sensor, the dietary record was revised together with the participant to complete information about quantities and form of food processing. A research dietitian checked the intake records and asked the participants for additional data if records were incomplete or implausible. In those incomplete records, the quantification of the amounts of food was made with the help of a visual method, consisting of a book with a series of photographs of food and dishes served, of different sizes, through which the participants could indicate in visual form the amount of food eaten [19].

The assessment of the dietary record was carried out using the software Dietowin® 7.1.

### Ethical considerations

The present study was reviewed and approved by the Clinical Research Ethics Committee from Galicia, Spain (CEIC2012–025). Written informed consent was obtained from each participant in the study, which conformed to the current Helsinki Declaration.

### Data analysis

Variables analyzed included clinical parameters (age, sex, body weight, height, body mass index, blood pressure, and waist measurement), meal composition (carbohydrates, fats, proteins, fiber, and calories) and glycemic postprandial responses.

The study period was defined from the start of dinner to 6 h later. The glycemic responses separated by sexes were compared according to intake of carbohydrates, fats, proteins and fiber.

Those subjects who met the prediabetes criteria according to the American Diabetes Association (fasting glucose ≥100 mg/dL and/or glycated hemoglobin ≥5.7%) were taken into account when performing the multivariable analysis [20].

### Statistical analysis

Generalized additive mixed-effects models (GAMMs) were used to describe changes over time in the glucose levels, which are modeled as penalized splines with random coefficients. As mixed models permit the incorporation of autocorrelation structures in residuals, GAMMs with the incorporation of autoregressive and moving average structures (as suggested by the exploratory analysis) were also investigated. Because of differences in behaviors of males and females, to assess changes in glucose levels, a nonlinear mixed-effects model was performed separately for each gender with the following structure:

$$ g\left({\mu}_{ij}\right)={X}_i^{\ast}\theta +{f}_i\left({x}_{1i}\right)+{f}_i\left({x}_{2i}\right)+{f}_i\left({x}_{3i},{x}_{4i}\right)+\dots +{\varepsilon}_{ij}+{Z}_{ij}{\alpha}_i, $$

where *μ*_*ij*_ ≡ *E*(*Y*_*ij*_), *Y*_*ij*_ is the response variable (glucose), ${X}_i^{\ast }$ is the arrow in the matrix for the parametric component model, *θ* is the corresponding parameter vector, the *f* (*x*)´s are smooth functions of the covariates, *ε*_*ij*_ are *N* (0, *σ*^2^) measurement errors, and *Z*_*ij*_*α*_*i*_ are *N* (0, *D*(*σ*^2^)) random effects [21].

All analyses were performed in R (version 3.4.3) using the packages “mgcv”, “nmle” and “gamm4.” These packages are freely available in R project (cran.r-project.org).

## Results

A total of 150 subjects (75 men and 75 women) with a complete continuous glucose monitoring were included. Those individuals for whom the CGM was incomplete (whether by sensor signal loss during the test or lack of capillary blood glucose data) were previously excluded. Among this initial sample, participants who had an unreliable dietary record were not included. Finally, 148 individuals (51% women) successfully completed all study procedures. Clinical parameters are detailed in Table 1.

**Table 1:** Clinical characteristics (mean ± standard deviation)

	Women (*n* = 75)	Men (*n* = 73)
Age (years)	50 ± 14	46 ± 14
Height (cm)	157 ± 6	172 ± 7
Weight (kg)	70 ± 11	85 ± 17
Body Mass Index (kg/m^2^)	28.4 ± 5	29.0 ± 5
Waist measurement (cm)	90 ± 13	97 ± 14
Systolic blood pressure (mm Hg)	129 ± 17	130 ± 12
Diastolic blood pressure (mm Hg)	77 ± 8	81 ± 8
HbA1c (%)	5.4 ± 0.4	5.4 ± 0.3
Blood glucose (mg/dL)	89 ± 11	92 ± 11
Subjects with Prediabetes (%)	32.0	28.8

A total of 888 dinners were analyzed (6 dinners for each individual). The mean energy intake at dinner was 680 ± 385 kcal. The intake was higher in males than in females (824 vs 531 kcal). Macronutrient distribution was similar in both sexes (19% proteins, 44% carbohydrates and 37% fats in men; 20% proteins, 44% carbohydrates and 36% fats in women). There were no significant differences in the amount of fiber between men and women (5.5 g vs 4.5 g).

The average duration of all CGM was 5.8 days (139.2 h), which means 1670 individual interstitial measurements. The mean blood glucose level before starting dinner intake was 106 mg/dL in women and 100 mg/dL in men. The mean glycemia measurements of each hour during the observation period (6 h) are shown in Table 2.

**Table 2:** Mean glycemia during the postprandial period

Mean glycemia (mg/dL)		
Time (min)	Women	Men
0	106	100
60	122	119
120	118	114
180	113	111
240	108	107
300	105	104
360	102	102

The percentage of values over the range of normal glycemia (> 140 mg/dL) in the postprandial period analyzed (6 h) was 9.7% in women and 7.1% in men.

**Fig. 1:**
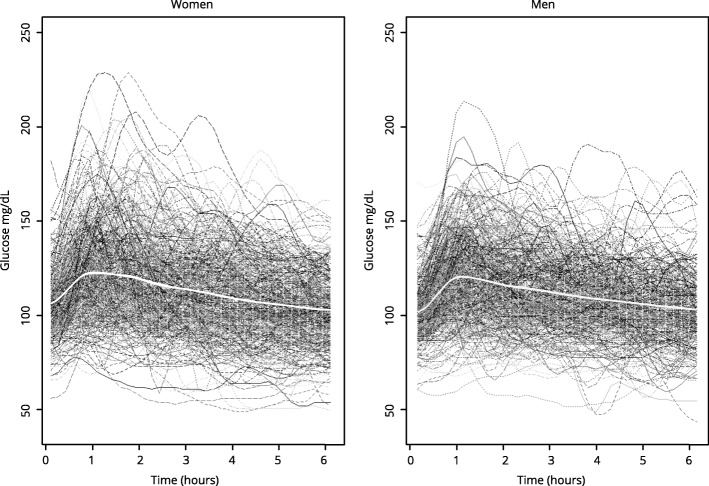
Glycemic response curves. Total glycemic response curves (mg/dL glucose) of the population sample (separated by sex) after dinner (6 h – postprandial period). The white line in the middle represents the mean

Glycemic response curves separated by sexes are shown in Fig. 1.

### Glycemic response in women

Those women who started with higher blood glucose levels before analyzed meal and those who had prediabetes presented a statistically higher postprandial glycemic response (Tables 3 and 4).

**Table 3:** Multivariable analysis: Postprandial glycemic response in women

	Coefficient	Standard Error	*p* value
Basal glycaemia (mg/dL)	0.0060	0.0003	0.0000
Carbohydrates (g)	0.0005	0.0002	0.0094*
Fiber (g)	−0.0028	0.0013	0.043**
Prediabetes (yes)	0.0692	0.0238	0.0037

The data in the table show the variables whose effect on the postprandial glycemic curve reached statistical significance

**Fig. 2:**
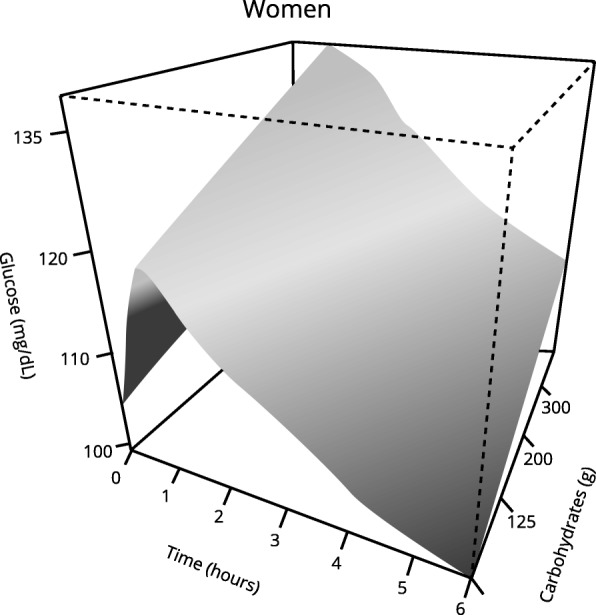
Effect of carbohydrates on the postprandial glycemic curve over time in women. Significantly different glycemic response was observed in those women who consumed more carbohydrates. *p* < 0.05 using generalized additive mixed-effects models (GAMMs)

*See Fig. 2 for a better understanding

**Fig. 3:**
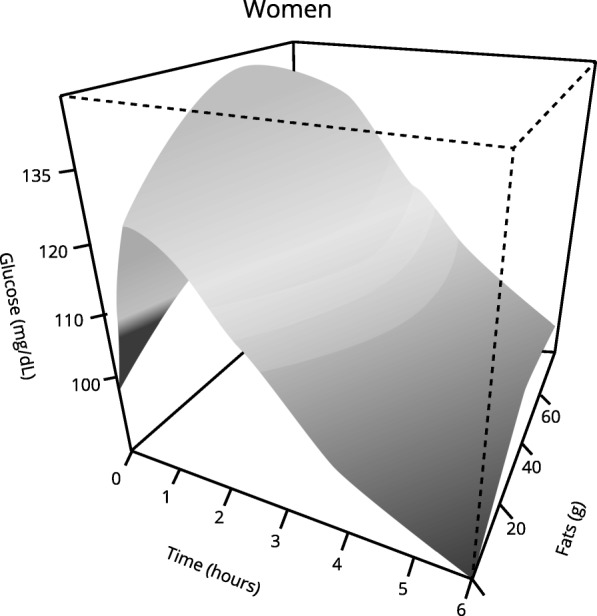
Effect of fats on the postprandial glycemic curve over time in women. Significantly different glycemic response was observed in those women who consumed more fats. *p* < 0.05 using generalized additive mixed-effects models (GAMMs)

** See Fig. 3 for a better understanding

**Table 4:** Multivariable analysis: Postprandial glycemic response in women

	Edf	*p* value
Time (min)	8.901	0.0000
Fats (g)	1.731	0.04*

**Fig. 4:**
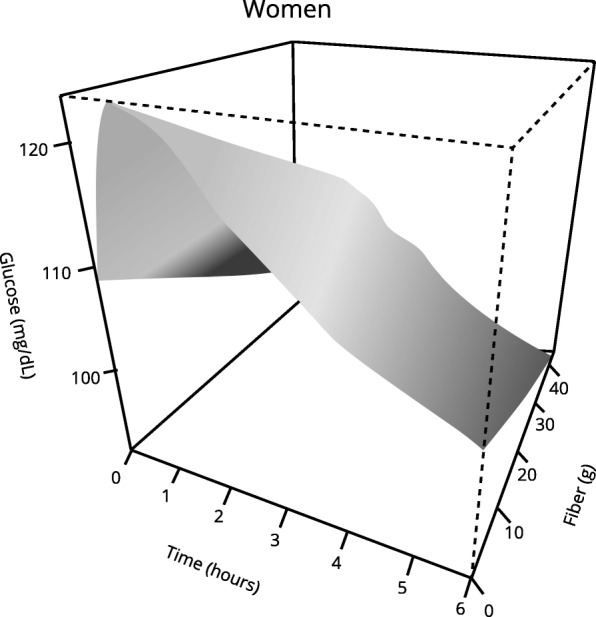
Effect of fiber on the postprandial glycemic curve over time in women. Significantly different glycemic response was observed in those women who consumed more fiber. *p* < 0.05 using generalized additive mixed-effects models (GAMMs)

*See Fig. 4 for a better understanding

edf: estimated degrees of freedom

*R*^2^ = 0.38

In relation to the nutrients analyzed, we found that carbohydrates, lipids, and fiber affect the postprandial glycemic response in women, although in different ways.

With regard to the influence of carbohydrates on postprandial glycemia, we observed that higher intakes correspond to a significantly higher glycemic response (Tables 3 and 4, Fig. 2). Women who ingested more carbohydrates at dinner had a higher glycemic peak, and they took longer to return to basal glycemic values (Fig. 2).

With regard to the effect of fats, women who ingested more lipids at dinner had longer lasting high glucose values, and they took longer to return to pre-meal glycemia. As fat intake was higher, a “flattening” of the postprandial glycemic curve was observed (Table 3 and 4, Fig. 3).

With respect to fiber intake, a significantly lower glycemic response was observed in the group of women whose fiber intake at dinner was higher (Tables 3 and 4, Fig. 4). Women who consumed a higher amount of fiber at dinner showed a lower postprandial glycemic peak as compared with those who hardly took any fiber.

**Fig. 5:**
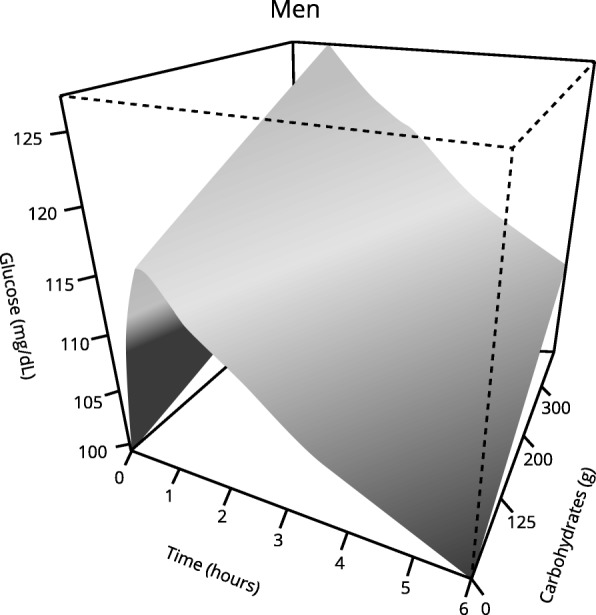
Effect of carbohydrates on the postprandial glycemic curve over time in men. Significantly different glycemic response was observed in those men who consumed more carbohydrates. *p* < 0.05 using generalized additive mixed-effects models (GAMMs)

### Glycemic response in men

As in the group of women, men who had a higher interstitial glucose values before dinner showed a higher postprandial glycemic response (Tables 5 and 6).

**Table 5:** Multivariable analysis: Postprandial glycemic response in men

	Coefficient	Standard Error	*p* value
Basal glycaemia (mg/dL)	0.0064	0.0004	0.0000
Carbohydrates (g)	0.0003	0.0001	0.0186*
Fibra (g)	−0.0004	0.0011	0.7157
Prediabetes (yes)	0.0254	0.0232	0.2726

The data in the table show the variables whose effect on the postprandial glycemic curve reached statistical significance

*See Fig. 5 for a better understanding

**Table 6:** Multivariable analysis: Postprandial glycemic response in men

	Edf	*p* value
Time (min)	8.932	0.0000
Fats (g)	1.000	0.553

edf: estimated degrees of freedom

*R*^2^ = 0.33

In relation to effects of nutrients analyzed, there were no significant differences in glycemic responses related to the amount of fats, proteins or fiber. Only intake of carbohydrates showed a significant influence on postprandial glucose values. Thus, subjects who consumed a higher quantity of carbohydrates at dinner showed a greater statistically significant glycemic response (Tables 5 and 6, Fig. 5).

## Discussion

The present study shows that CGM is a tool that allows a better understanding of glycemic response to meals. One of the advantages of using this system is that volunteers were in free-living conditions and it is likely to reflect a more representative situation than a laboratory setting. There are few studies that allow participants to maintain their usual routine of meals and exercise. Most studies are conducted under controlled conditions, and in some of them, the studies are done under hospital admission.

The average duration of the monitoring period in our study was 5.8 days. Previous studies were carried out with shorter monitoring periods [22, 23].

We analyzed 148 individuals. Thus far, published works used smaller populations that rarely exceeded 100 individuals [22–25].

In relation to the percentage of time in the hyperglycemia range, the results of previous works were variable, ranging from 1.8% [24] to 13% [21], and always referring to the analysis of 24 h a day, not just the postprandial period.

Carbohydrate is the dietary component that primarily influences blood glucose response. Previous studies have confirmed that the total intake of carbohydrates, either as a “snack” or main meal, can consistently predict the concentrations of glucose in the postprandial period. Wolever et al. concluded that carbohydrate content and glycemic index together explained about 90% of the variation in the glycemic response [26].

In the present study, the results showed that the glycemic response was significantly higher in those individuals who consumed a greater amount of carbohydrates at dinner, in both sexes.

The influence of fats on the postprandial glycemic profile was different in women compared with men. In the group of women, the glycemic response was significantly different in those who consumed more fat. In more detail, it a “flattening” of the glycemic curve was observed.

In a study in which 11 healthy subjects were selected to evaluate the effect of adding fat to a meal, the findings are consistent with ours. Thus after intake of a high-fat meal, both the glycemic response (incremental area under the curve) and the glycemic peak were lower [27].

In one of the last published works that refer to this issue, the authors also observed the same effect. In that study, the glycemic response (capillary blood glucose up to 3 h) was evaluated in a sample of 12 healthy subjects after adding peanut oil to a white rice meal. A significant decrease occurred in the incremental area under the curve and glycemic peak [28].

The amount of dietary fat is one of the main factors that modify the glycemic response after ingestion of mixed meals. This is probably because of a delay in absorption of glucose in the small intestine, secondary to inhibition of gastric emptying produced by the presence of fat. Furthermore, the fat content of meals produces an increased incretin secretion, which also implies a reduction of the glycemic response [29].

Another explanation for flattened blood glucose in women could be their lower muscle mass and higher total body fat that will produce a lower glucose uptake in the muscle cells. As women have lower muscle mass they will have lower lipoprotein lipase activity and a less effective triglycerides mechanism removal [30]. Besides, lipid accumulation in skeletal muscle impairs insulin signaling and then contributes to the flattened blood glucose response [31].

The differences found between men and women could also be related with gonadal hormones. Women and men of similar age have no differences in insulin sensitivity but after menopause there is a decline in insulin sensitivity and an increase in fat mass [32]. A significant amount of women in our study are probably in the menopause period. That circumstance could have influenced the glucose response to fat intake. Also estradiol has a positive influence on insulin sensitivity and beta cells pancreatic function that could be loss after menopause [33].

In relation to the effect of fiber, in our group of women, a significantly lower postprandial glycemic response was observed in those who consumed more fiber at dinner. By contrast, in the group of men, no different glycemic responses were found depending on fiber intake at dinner.

Potter analyzed blood glucose (3 h) after eating 4 meals with the same amount of macronutrients and different fiber content. The biggest difference was observed between 30 and 60 min postprandially, where food with less fiber produced a higher peak. At 3 h after ingestion of food with more fiber, glucose was lower [34]. Brynes et al. found that fasting blood glucose, mean blood glucose, and the AUC were lower after increasing fiber intake for a week (13.5 g vs 22.3 g) in the diet of 9 healthy subjects. The analyzed period was 24 h, and the participants used the CGM system [14]. Similar results were obtained by Sun et al., who compared the glycemic response after ingestion of two meals with same amount of carbohydrates and different fiber content (rice with and without vegetables) [28].

Dietary fiber has also been reported to modify glycemic response. This effect may be due to the delay produced by fiber in the following processes: starch digestion in the stomach, transition of stomach contents into the duodenum, hydrolysis of the polysaccharides in the duodenum, and absorption time of monosaccharides [35]. In addition, fiber intake is associated with increased sensitivity to insulin, resulting in lower levels of plasma insulin and lower blood glucose levels [36].

In females, those who are in a prediabetes situation had a significantly higher postprandial glucose levels. The same fact was observed in the group of males, but without reaching statistical significance. However, the effect of nutrients on the postprandial glycemic response was not modified by this factor in either of the two groups.

In sum, the postprandial glycemic response in women was different from that of men. The causes of the differences in glucose control are not clearly understood, although gender-related differences in body fat distribution and hormones as well as slower absorption in women may contribute to the observed gender dimorphism [37, 38].

Several epidemiological studies revealed sex-specific differences during the oral glucose tolerance test (OGTT), such as a higher prevalence of glucose intolerance in females. To date, the gender-related differences in the incidence of impaired glucose tolerance (IGT) are still under debate. Sicree et al. [39] reported higher IGT prevalence in Australian women in comparison with men and ascribed this observation to differences in height between both genders. In addition, they supposed that taller persons (mostly males) have more muscle mass, which is the major tissue involved in glucose uptake.

Anderwald et al. [37] found that females showed lower fasting endogenous glucose production and decreased plasma glucose levels during the early course of the OGTT but higher plasma glucose concentrations from 120 to 180 min. They also observed that the glucose absorption rates were higher in males in the initial phase of the OGTT and elevated in females in the final part of the 180 min OGTT. The best-suited value to describe OGTT glucose absorption velocity is the half-life of glucose in the gut, which was prolonged in females in comparison with males. Thus, these differences in glucose absorption could serve to explain higher glucose concentrations at the end of the OGTT in females [37].

Furthermore, glucose levels also depend on other variables, such as insulin sensitivity, insulin secretion, hepatic glucose absorption and release of glucagon and incretins. Some of these variables were shown to differ between men and women [40].

There are some limitations in our study. It should be taken into consideration that in the dietary record individuals may forget some information or underestimate the intake. In addition, physical activity could affect the glycemic response and it was not taken into account when performing the analysis, because it was not the objective of the study. Otherwise, the fact of having done the study in free living conditions and in a large number of subjects, gives an external validity and the results could be generalized to other groups of populations.

Future studies are necessary to study in depth how gender affects the postprandial glycemic responses. Increase knowledge of glucose response to meals can contribute to better management of diseases related to glucose metabolism.

## Conclusions

We conclude that glycemic response to meals is different in men and women. Meal carbohydrates are the main determinants of elevated postprandial glucose levels and the glycemic response. In women, fat content of meal is linked to higher postprandial glucose values and a flattening of postprandial glycemic response; and fiber produces a decreased glucose response. No effect of protein content was observed in either sex.

## Data Availability

All data generated or analysed during this study are included in this published article.

## Abbreviations

CGM: Continuous Glucose Monitoring
GAMM: Generalized Additive Mixed-effects Models
IGT: Impaired Glucose Tolerance
OGTT: Oral Glucose Tolerance Test
